# Mechanism of dexmedetomidine preconditioning on spinal cord analgesia in rats with functional chronic visceral pain

**DOI:** 10.1590/acb370203

**Published:** 2022-05-02

**Authors:** Jun Li, Huizhong Tang, Weifeng Tu

**Affiliations:** 1 Graduate student. Southern Medical University – Guangzhou, China.; 2 MD. Department of Radiology – People’s Hospital of Guangxi Zhuang Autonomous Region – Nanning, China.; 3 MD. Department of Anesthesiology – General Hospital of Southern Theatre Command – Guangzhou, China.

**Keywords:** Dexmedetomidine, Functional Chronic Visceral Pain, Toll-Like Receptor 4, Analgesia, Electrophysiological Indexes

## Abstract

**Purpose:**

To analyze the effect and mechanism of dexmedetomidine (DEX) analgesia pretreatment on functional chronic visceral pain in rats.

**Methods:**

Rats were divided into six groups: W1, W2, W3, W4, W5, and W6. The behavioral changes and electrophysiological indexes of rats in each group before and after DEX treatment were detected.

**Results:**

The levels of abdominal withdrawal reflex (AWR) in W5 and W6 groups were significantly lower than those in group W3, while the levels of thermal withdrawal latency (TWL) and mechanical withdrawal threshold (MWT) were significantly higher than those in group W3 (p *<* 0.05). The electromyographic signals of W1, W5, and W6 groups showed little fluctuation, while those of groups W2, W3, and W4 showed obvious fluctuation. TLR4 mRNA expression, IRF3, P65, and phosphorylation levels in W4, W5, and W6 groups were significantly lower than those in group W2 (p *<* 0.05).

**Conclusions:**

Dexmedetomidine epidural anesthesia pretreatment could significantly inhibit visceral pain response in rats with functional chronic visceral pain, and its mechanism was related to the activation of TLR4 in spinal dorsal horn tissue of rats and the activation inhibition of IRF3 and P65 in the downstream key signals.

## Introduction

Visceral pain is mainly caused by visceral dysfunction^1^. The common causes include organ inflammation and mechanical traction. Angina pectoris, myocardial infarction, arrhythmia, and other diseases that can cause myocardial insufficiency lead to visceral pain^1^^,^ 2. Visceral pain is characterized by slow and continuous pain, and unclear positioning. It is often accompanied by pain, and emotional or defensive response, resulting in nausea, fever, discomfort, and pain^3^. Relevant data show that more than 25 million patients enter the emergency room due to pain and 2.5 million patients are hospitalized due to pain every year in the United States. Among them, chest, abdominal or pelvic acute and chronic diseases, including tumors, are one of the most important causes of pain^4^^,^ 5. With the development of economy and the deepening of aging, the number of people suffering from visceral pain in Chinese society is also gradually increasing, and the incidence of young groups is prominent. More than 10% of children have a history of abdominal pain, and about half of these children are likely to continue to experience abdominal pain and similar problems after more than 20 years^6^. In addition, inadequate perioperative management of visceral pain and the use of high-dose opioids can also induce and lead to visceral pain sensitization and chronic pain, affecting the quality of life of patients after surgery.

There is no specific method for the treatment of visceral pain. Dexmedetomidine (DEX) is a highly selective Î±-2 receptor adrenergic agonist, which has the characteristics of antagonistic sympathetic tension, sedation, and analgesia^7^^,^ 8. It has been widely used in clinical analgesia, such as enhancing the role of local anesthetics and prolonging the block time, protecting nerves and organs, preventing and reducing postoperative delirium, and preventing postoperative chills^7^–^9^. Dexmedetomidine can be administered through continuous intravenous infusion, intramuscular injection, oral administration, nasal drip or buccal mucosa. However, it has significant liver first-pass elimination effect, and the oral bioavailability is only 16%^10^. A large number of clinical studies have found that intraoperative DEX anesthesia can produce good synergistic effect with other sedative and analgesic drugs, and can significantly reduce the use of other sedative and analgesic drugs. Dexmedetomidine is clinically used, and the main adverse reactions and side effects are hypotension and bradycardia^11^. Rapid intravenous infusion of DEX can lead to increased initial blood pressure and decreased heart rate reflex, which is more common in young healthy patients. Most of the current studies focus on the use of DEX intravenous anesthesia, and there is little analysis on the mechanism of physical pain and visceral pain^12^^,^ 13.

To sum up, in this study, Sprague Dawley (SD) neonatal rats were used as the research sample. The preparation of irritable bowel syndrome-like functional chronic visceral pain rat model, according to different treatment programs are divided into groups W1, W2, W3, W4, W5, and W6. The behavioral changes and electrophysiological indexes were detected in each group before and after DEX treatment, so as to comprehensively evaluate the application mechanism of DEX anesthesia preconditioning in the treatment of functional chronic visceral pain.

## Methods

### Experimental animals

The Animal Care and Use Committee of Qingdao Municipal Hospital, School of Medicine Qingdao University, Qingdao approved the research protocol.

Fourteen clean-grade adult SD rats purchased from Liaoning Changsheng Biotechnology Co. Ltd. were fed in seven cages, one male and one female in each cage. The ambient temperature was maintained at about 27°C, and the normal circadian rhythm was maintained. They were free to eat, drink, and wait for pregnancy. The newborn rats were used as the research samples, and the feed and drinking water were replaced daily. In order to reduce the stimulation, the bedding was replaced once a week to maintain the clean and healthy living environment. During the experiment, rats were given humane care according to the 3R principles of animal experiments (reduction, refinement, and replacement).

### Preparation of rat model of irritable bowel syndrome-like functional chronic visceral pain

On the second day after birth, the young rats were separated from their mothers. At 9 am, the pups were placed in a new cage with the original feeding environment and transferred to anew room. The temperature setting was the same as the original feeding environment. After 5 h, the pups were put back to the original environment. This step lasted for 2 weeks.

Seven days after birth, the young rats were put into the model room at 9 am and wiped around the anus of the young rats by wet warm water with cotton swabs for two circles. The 3-mm diameter human vascular reconstruction balloon was inserted into the colon and rectum of the young rats from the anus, and the balloon was completely entered into the anus. The balloon was inflated and maintained for 1 min, and then the balloon was released. The above operation was repeated after 30 min. After each expansion, the young mice were sent to the mother rats immediately.

**Figure 1:**
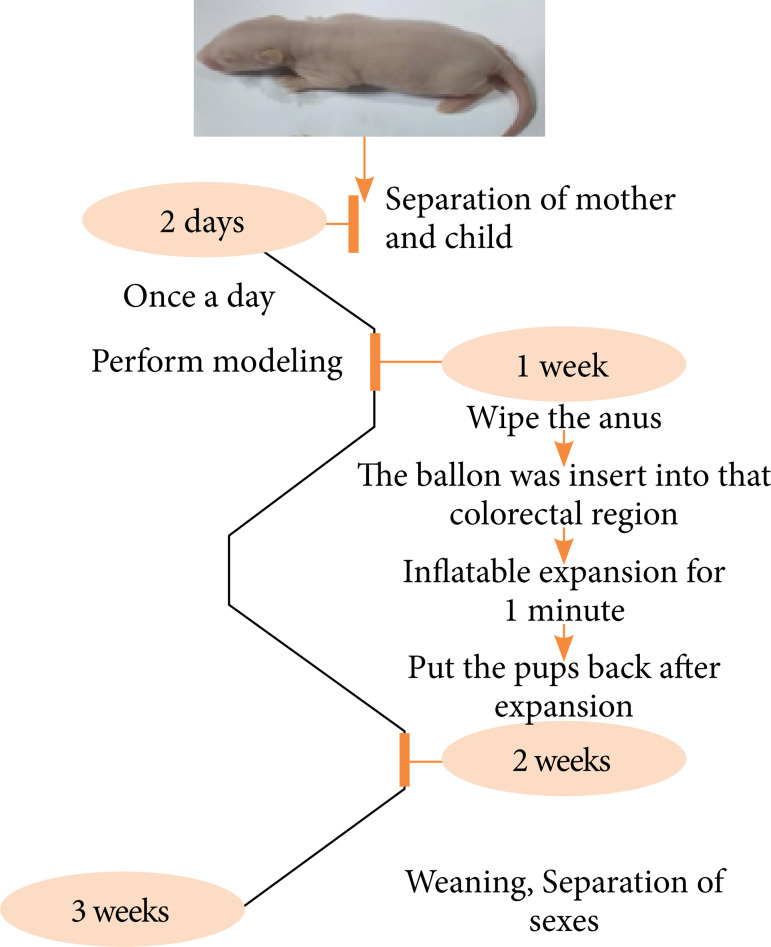
Flow chart of rat model of irritable bowel syndrome-like functional chronic visceral pain.

At the third week after birth, the young rats were weaned. The male and female rats were fed in separate cages. Flow chart of rat model of irritable bowel syndrome-like functional chronic visceral pain is shown in Fig. 1.

Model exclusion criteria^14^: (1) Rats with too much or too little weight in each cage; (2) Rats which died before the observation time point; (3) Elimination of inaccurate brain positioning. If the number of animals in each experimental group is less than the preset number due to the above exclusion factors, the number of animals shall be supplemented by the principle of random sampling.

### Animal grouping

Forty-two SD rats were divided into six groups with seven rats in each group. Group W1 as blank control, do not accept epidural DEX pretreatment nor epidural 5% dimethyl sulfoxide (DMSO) injection, and do not establish acute inflammatory visceral pain model in rats. Rats in Group W2 received epidural injection of 5 % 0.3 mL DMSO, but did not receive epidural DEX pretreatment. Group W3 rats were the chronic visceral pain model group, and were not given DEX pretreatment. Rats in Group W4 were given epidural injection of 0.3 mL 5% DMSO containing 6 μg DEX. Rats in Group W5 were given epidural injection of 0.3 mL 5 % DMSO containing 26 μg DEX. Rats in W6 group were given epidural injection of 0.3 mL 5 % DMSO containing 52 μg DEX.

### Determination of thermal withdrawal latency (TWL), mechanical withdrawal threshold (MWT), and abdominal withdrawal reflex (AWR)

Determination of AWR: After the rats were anesthetized, an angioplasty balloon with a diameter of 3mm and a length of 20mm was inserted into the anus. Entered the anus, it was fixed on the tail of the rats with adhesive tape. After the rats were awake, the stimulation intensity of colorectal dilatation included 80, 60, 40, and 20 mmHg. The colorectal distension stimulation lasted for 20 s with an interval of 5 min. Obvious lifting of the lower abdominal wall from the box bottom or obvious flattening of contraction were observed with the naked eye (AWR score was 3 points). The standard of AWR score was 0, and there was no significant behavioral change. When AWR score was 1, only simple head movement occurred in rats. When AWR score was 2 points, the abdominal muscles began to contract. When AWR score was 3 points, the lower abdominal wall of rats was lifted from the bottom of the box or significantly contracted. When AWR score was 4 points, the minimum pressure when the abdominal wall arched or with the body and pelvis bowed was the pain response threshold.

Determination of MWT: The rats were placed on a metal net covered with a transparent plexiglass cover. After the rats adapted to the environment (about 15 min, the combing and exploration activities of the rats basically disappeared), the center of the rat’s hind paw was stimulated with a standardized Von Frey test probe to make it bend into an S-shape for 4 ~ 6 s, and the foot retraction reaction of the rats was observed. A positive reaction was defined as a rapid foot retraction, or biting of the stimulated claw, in rats either during the time of stimulation or immediately upon removal of Von Frey needles. However, the foot contraction reaction caused by physical activity in rats was not regarded as a positive reaction. Each stimulation was repeated five times, and the interval between two consecutive measurements was 30 s. The threshold of 50% positive reaction in rats was determined by titration, that is, the probe stimulation pressure value (g) was the MWT of the rat.

Measurement of TWL: By using intensity adjustment to stimulate the hind paw of rats, the radiation was controlled below 20 s to avoid burns. Each measurement was repeated five times, with an interval of 3 min between two consecutive measurements, the minimum and maximum values were removed, and the final value was defined as the average value of residual values.

### Visceral motor response and mean arterial blood pressure measurement

Visceral motor response: After inhalation of ether anesthesia, the rats were fixed in the cage. They were connected to the biological function experimental system through silver electrode. The response of rat myoelectric signal diagram to colorectal dilation stimulation was recorded and analyzed by computer.

Mean arterial pressure (MAP)^15^: After rats were anesthetized with ether, their tail was heated by electric wind until the skin of tail became slightly red, and the tail sleeve noninvasive blood pressure tester was installed for measurement (the pressure pulse of the sphygmomanometer was sleeved to the proximal end of the tail of rats, and the high-sensitivity pulse transducer was placed in the middle and upper one-third of the tail. The surface of the transducer was aligned to the ventral side of the tail. After fixation, the balloon was used to monitor the systolic and diastolic blood pressure of rats through intermittent aeration and deflation, and the MAP was calculated after recording).

### Hematoxylin-eosin (HE) staining

The colon tissue and spinal dorsal horn tissue of rats were stained with HE. Specific steps were: The fixed tissue samples were performed for toluene, ethanol dewaxing, and hematoxylin staining for 5 min, washed by distilled water 3 s, washed by 1% hydrochloric acid ethanol for 3 s, washed by distilled water 30 s, dyed with 0.5% eosin for 3 min, washed with distilled water for 3 s, and washed with 80% ethanol for 2 s.

According to the results of HE staining, the damaged neurons were counted and HE score was used to reflect neurotoxicity, as follows: (1) Irregular neurons with 0–25%, 0 points; (2) Irregular neurons 26–50 %, 1 point; (3) Irregular neurons 51–75%, 2 points; (4) Irregular neurons 76–100%,3 points.

### Polymerase chain reaction (PCR) extraction

RNA was extracted from the dorsal horn of rat spinal cord with Trizol reagent (Invitrogen), and the level of messenger RNA (mRNA) was measured by light-cycle 480 real-time PCR system (Roche, Germany). Quantitative PCR was performed in reaction volume containing 20 μL SYBR Green PCR Master Mix. The transcript level (mean of control) was determined relative to the calibrator and normalized to the endogenous reference (2 ^– ΔΔCT^ method)^16^.

*Statistical methods*

The data of this study were analyzed by SPSS19.0 statistical software. The measurement data were expressed by mean ± standard deviation (x±s), and the counting data were expressed by percentage (%). Single factor analysis of variance was used for pairwise comparison. The difference was statistically significant (p *<* 0.05).

## Results

### Comparison of TWL, MWT, and AWR in rats

**Figure 2:**
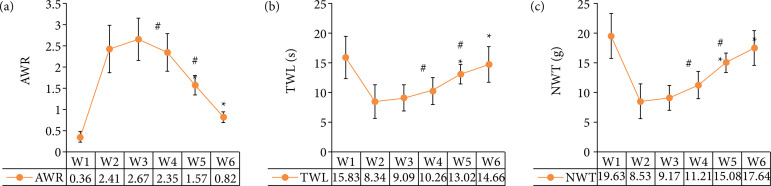
Comparison of **(a)** AWR, **(b)** TWL and **(c)** MWT in rats.

As shown in Fig. 2, the levels of AWR in W5 and W6 groups were significantly lower than those in group W3, while the levels of TWL and MWT were significantly higher than those in group W3 (p *<* 0.05). There was no significant difference in the levels of AWR, TWL, and MTL between group W4 and Group W3 (p*>* 0.05). The levels of AWR in W6 group were significantly lower than those in groups W4 and W5, while the levels of TWL and MWT were significantly higher than those in groups W4 and W5 (p *<* 0.05).

* indicates that the difference is statistically significant compared with group W3 (p < 0.05).# compared with W6 group, the difference is statistically significant (p < 0.05).

### Comparison of electromyogram (EMG) signals in rats

**Figure 3:**
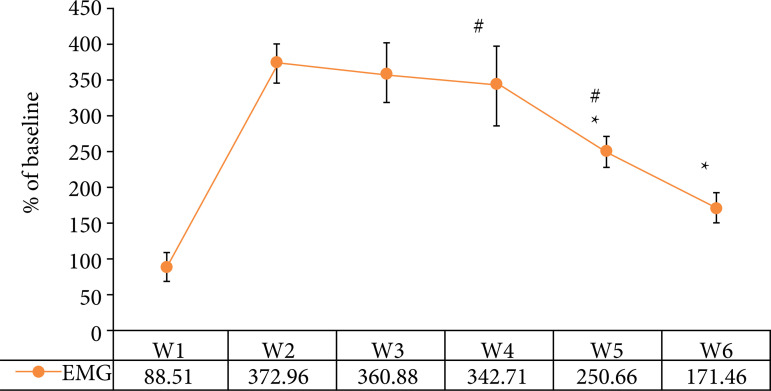
Comparison of rat EMG signals.

**Figure 4:**
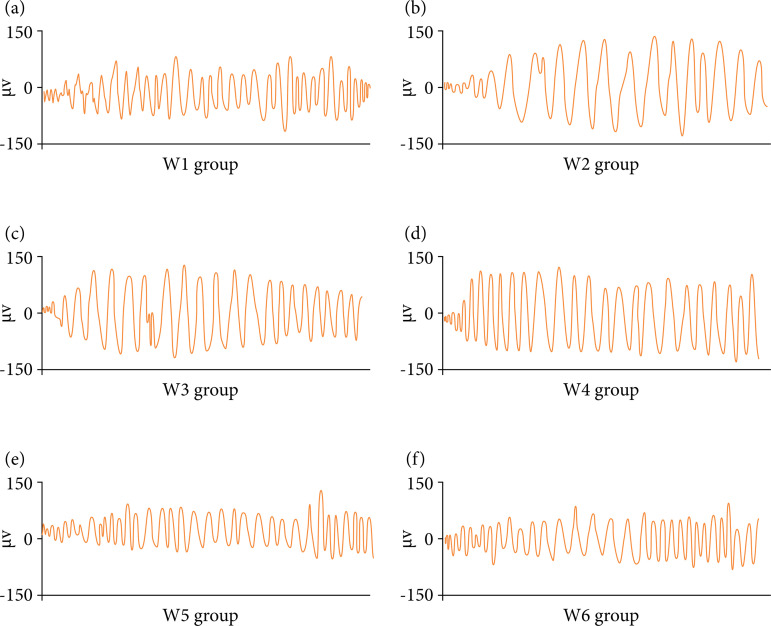
EMG of rats.

As shown in Fig. 3, the EMG signal levels of rats in W5 and W6 groups were significantly lower than those in group W3, and the difference was statistically significant (p *<* 0.05). There was no significant difference in EMG signal level between group W4 and Group W3 (p *>* 0.05). The level of EMG signal in W6 group was significantly lower than that in groups W4 and W5 (p *<* 0.05). Figure 4 shows the EMG signal diagram of rats. The fluctuation amplitude of EMG signal in W1, W5, and W6 groups is small, while the fluctuation amplitude of EMG signal in groups W2, W3, and W4 is obvious.

* indicates that the difference is statistically significant compared with the group W3 (p *<* 0.05).# indicates that the difference is statistically significant compared with the group W6 (p *<* 0.05).

(a) EMG of rats in group W1; (b) EMG of rats in group W2; (c) EMG of rats in group W3;(d) EMG of rats in group W4; (e) EMG of rats in group W5; (f) EMG of rats in group W6.

### Hematoxylin-eosin staining of rat colon

**Figure 5:**
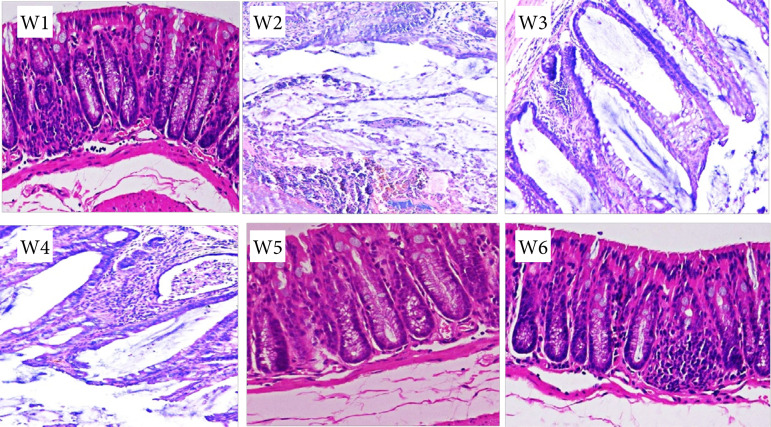
HE staining of rat colon.

As shown in Fig. 5, the rats in W1, W5, and W6 groups have no obvious pathological changes, such as intestinal cavity expansion and adhesion with surrounding tissues, no obvious tissue damage, no neutrophil infiltration, and no obvious edema in stroma. The HE staining of rats in groups W2, W3, and W4 shows obvious damage and neutrophil infiltration.

### Comparison of noninvasive blood pressure in rats

**Figure 6:**
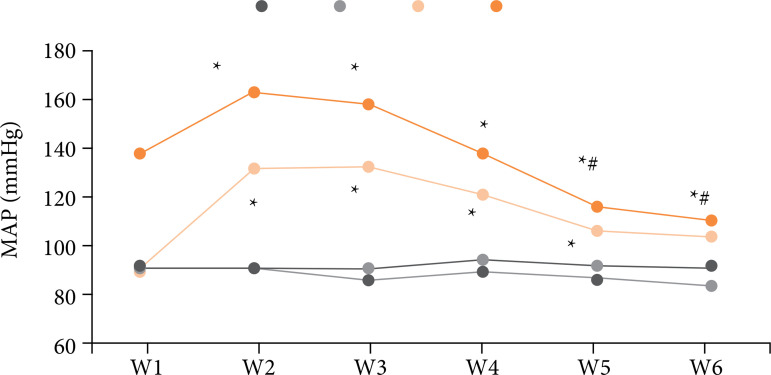
Noninvasive blood pressure of rats (1–4 are conditions 5 min after ether anesthesia, 5 min after administration intervention, 10 min after modeling, and after AWR stimulation, respectively).

As shown in Fig. 6, there was no significant difference in MAP levels between W2, W3, W4, W5, and W6 groups and group W1 5 min after ether anesthesia and 5 min after administration intervention (p *>* 0.05). Ten minutes after modeling, the MAP levels of rats in W2, W3, W4, W5, and W6 groups were significantly higher than those in group W1 (p *<* 0.05). After AWR stimulation, the MAP levels of W5 and W6 rats were significantly lower than those of group W1 (p *<* 0.05). The MAP levels of W5 and W6 rats were significantly lower than that of group W4 (p *<* 0.05).

* indicates that the difference is statistically significant compared with group W1(p *<* 0.05) * compared with group W4, the difference is statistically significant (p *<* 0.05).

### Neurotoxicity of rat spinal cord

**Figure 7:**
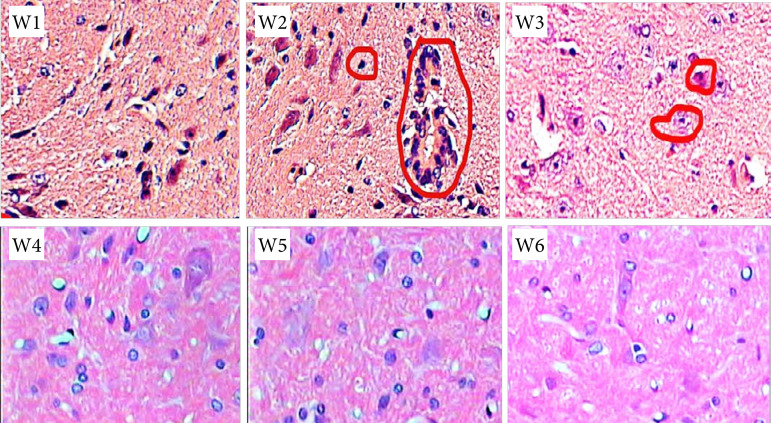
Staining of rat spinal dorsal horn.

**Figure 8:**
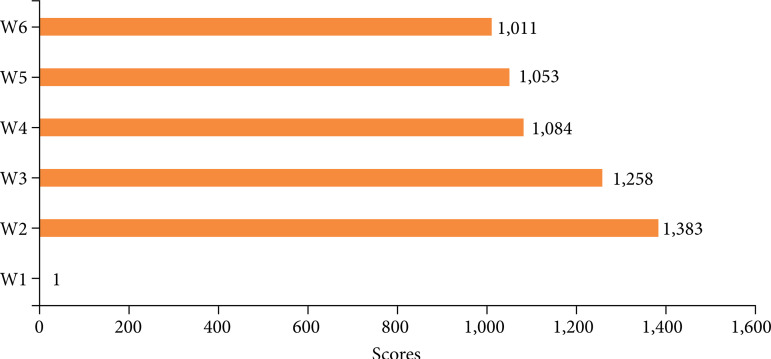
Comparison of HE staining scores of rat spinal cord neurons.

Figure 7 shows the HE staining of the spinal dorsal horn of each rat, showing blue nuclei, in which the calcium salt granules and cartilage matrix were dark blue, and the mucus was gray blue. Pink cytoplasm, in which eosinophilic particles were bright red, red blood cells were orange. The HE staining scores of spinal neurons in the W4, W5, and W6 groups were not significantly different from those in the control group (p *>* 0.05) (Fig. 8). There was no significant difference in HE staining scores of spinal cord neurons between the W4, W5 and W6 groups (paired comparison) (p *>* 0.05).

* indicates that the difference is statistically significant compared with group W3 (p *<* 0.05).

### mRNA expression of rat TLR4

**Figure 9:**
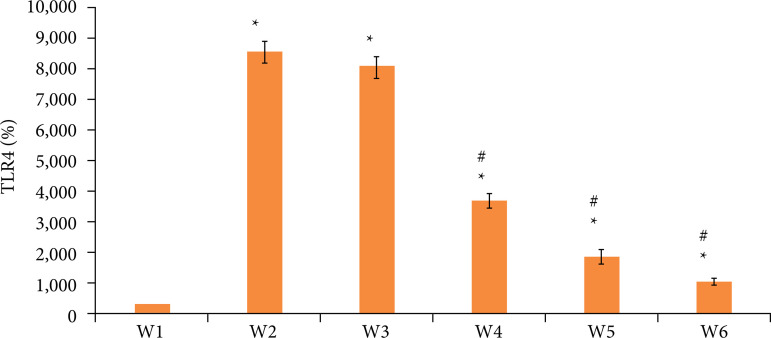
Comparison of TLR4 mRNA expression levels in rats.

As shown in Fig. 9, the mRNA expression of TLR4 in group W1 was significantly lower than that in W2, W3, W4, W5, and W6 groups (p < 0.05). The mRNA expression of TLR4 in W4, W5, and W6 groups was significantly lower than that in group W2 (p < 0.05).

* indicates that the difference is statistically significant compared with group W1 (p < 0.05)# indicates that there is significant difference in group W2 (p < 0.05).

### Expression of IRF3, P65, and phosphorylation levels in rats

**Figure 10:**
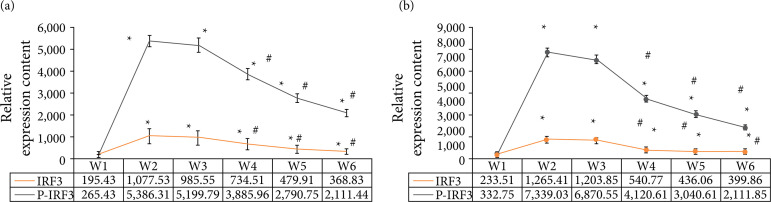
Expression of IRF3, P65, and phosphorylation levels in rats (A shows IRF3 and phosphorylation levels. B is P65 and phosphorylation level).

As shown in Fig. 10, the expression levels of IRF3, P65, and phosphorylation in group W1 were significantly lower than those in W2, W3, W4, W5, and W6 groups (p < 0.05). The expressions of IRF3, P65, and phosphorylation in W4, W5, and W6 groups were significantly lower than those in group W2 (p < 0.05).

* indicates that the difference is statistically significant compared with group W1 (p < 0.05) # indicates that there is significant difference in group W2 (p < 0.05).

## Discussion

Functional abdominal pain syndrome, also known as chronic functional abdominal pain, is mainly characterized by chronic visceral pain, accompanied by a certain degree of decline in daily activity^17^. Although the disease will not endanger the patient’s life, it will have a serious impact on the patient’s quality of life^17^^,^ 18. Therefore, it has become a research hotspot all over the world to find an analgesic drug with clear effect, safety and effectiveness, low tolerance and low addiction, or a nonopioid analgesic drug that can enhance the analgesic effect of opioids and inhibit the tolerance and dependence of opioids^19^.

In this study, the rat model of irritable bowel syndrome like functional chronic visceral pain was prepared with SD neonatal rats and young rats as research samples, and the behavioral changes and electrophysiological indexes of rats in each group before and after DEX treatment were detected. Firstly, it was found that the levels of AWR in W5 and W6 groups were significantly lower than those in Group W3, while the levels of TWL and MWT were significantly higher than those in group W3 (p < 0.05), which was similar to the research results of Palmer and Baysinger^20^, indicating that the treatment of medium and high dose DEX can effectively inhibit the pain sensitivity and animal behavior of rat model. The levels of AWR in W6 group were significantly lower than those in groups W4 and W5, while the levels of TWL and MWT were significantly higher than those in Groups W4 and W5 (p < 0.05). The results showed that high-dose DEX had the best inhibitory effect. The level of EMG signal in W6 group was significantly lower than that in groups W4 and W5, and the difference was statistically significant (p < 0.05), indicating that high-dose DEX had the best inhibitory effect on electrophysiological response in rats^21^. From the EMG signal diagram of rats, the fluctuation amplitude of EMG signals in W1, W5 and W6 groups was small, while the fluctuation amplitude of EMG signals in Groups W2, W3, and W4 was obvious, which was consistent with the results of quantitative data.

After AWR stimulation, the MAP level of rats in W5 and W6 groups was significantly lower than that in group W1, and the MAP level of rats in W5 and W6 groups was significantly lower than that in group W4 (p < 0.05), which was similar to the research results of Zhou *et al*.^22^, indicating that medium and high doses of DEX can effectively inhibit the arterial pressure level of rats, and the effect of high doses of DEX was the best.

Comparing the HE staining scores of spinal cord neurons in rats, it was found that there was no significant difference in the HE staining scores of spinal cord neurons in W4, W5, and W6 groups compared with the control group (p*>* 0.05). There was no significant difference in HE staining scores of spinal cord neurons in W4, W5, and W6 groups (paired comparison) (p*>* 0.05), indicating that low, medium and dose DEX had no significant effect on spinal cord nerves in rats. The mRNA expression of TLR4 in W4, W5, and W6 groups was significantly lower than that in group W2 (p < 0.05). The expressions of IRF3, P65, and phosphorylation in W4, W5, and W6 groups were significantly lower than those in group W2 (p < 0.05). It comprehensively shows that DEX epidural preconditioning can inhibit the activation of TLR4 and the activation of downstream key signals IRF3 and P65 in rat spinal dorsal horn, so as to play the role of antichronic visceral pain, among which the analgesic effect of high-dose DEX is the best^23^.

## Conclusions

In this study, SD neonatal young rats were used as research samples to prepare the rat model of irritable bowel syndrome-like functional chronic visceral pain, and the behavioral changes and electrophysiological indexes of rats in each group before and after DEX treatment were detected. The results showed that DEX epidural anesthesia pretreatment could significantly inhibit visceral pain sensitivity in rats with functional chronic visceral pain, and its mechanism was related to the activation of TLR4 in spinal dorsal horn and the activation and inhibition of downstream key signals IRF3 and P65. However, the division of anesthetic dose in this study is relatively rough, there is no further subdivision form, and the data of rat pain model is less, so it is necessary to make further experimental analysis in the future. In conclusion, the results of this study provide a data reference for the application of DEX anesthesia in clinical analgesic treatment.
